# A new model based inflammatory index and tumor burden score (TBS) to predict the recurrence of hepatocellular carcinoma (HCC) after liver resection

**DOI:** 10.1038/s41598-022-12518-5

**Published:** 2022-05-23

**Authors:** Jianhua Wang, Zeguo Chen, Liheng Wang, Sijia Feng, Qixuan Qiu, Dongdong Chen, Nianfeng Li, Yao Xiao

**Affiliations:** 1 Department of General Surgery, Xiangya Hospital, Central South University, Changsha, 410008 China; 2 National Clinical Research Center for Geriatric Disorders, Xiangya Hospital, Central South University, Changsha, 410008 China; 3 International Joint Research Center of Minimally Invasive Endoscopic Technology Equipment and Standards, Changsha, 410008 China

**Keywords:** Cancer, Biomarkers, Health care, Medical research, Risk factors

## Abstract

To establish a model based on inflammation index and tumor burden score (TBS) to predict recurrence of hepatocellular carcinoma (HCC) after liver resection. A retrospective study was performed on 217 patients who diagnosed HCC underwent liver resection at Xiangya Hospital Central South University from June 1, 2017 to June 1, 2019. According to the receiver operating characteristic (ROC) curve, the optimal cut-off value of inflammatory index and the TBS was determined by the Youden index. Prediction performance was compared by the area under the receiver operating characteristic curve (AUC). Cox regression analysis was used to determine the risk factors for the recurrence of HCC after liver resection. According to the independent risk factors of the patients, a prediction model for HCC was established based on inflammation index and tumor burden score (TBS).The prediction performance of the model was compared with single index (TBS group and NLR group) and traditional HCC stage models (TNM stage and BCLC stage). MLR = 0.39, NLR = 2.63, PLR = 134, SII = 428 and TBS = 8.06 are the optimal cut-off values. AUC of SII, PLR, NLR, MLR and TBS were 0.643, 0.642, 0.642, 0.618 and 0.724respectively. MVI (*P* = 0.005), satellite nodule (*P* = 0.017), BCLC B-C stage (*P* = 0.013), NLR > 2.63 (*P* = 0.013), TBS > 8.06 (*P* = 0.017) are independent risk factors for the recurrence of HCC after liver resection. According to this study, the optimal inflammatory index NLR combined with TBS was obtained. The AUC of NLR–TBS model was 0.762, not only better than NLR group (AUC = 0.630) and TBS group (AUC = 0.671), also better than traditional BCLC (AUC = 0.620) and TNM (AUC = 0.587) stage models. Interestingly, we found that NLR and TBS should be good prognostic factor for recurrence of HCC after liver resection. The NLR–TBS model based the best inflammatory index (NLR) and TBS have a better prediction performance and the prediction performance of NLR–TBS model not only better than NLR group and TBS group, but better than BCLC and TNM stage models.

## Introduction

Primary liver cancer is the sixth most commonly diagnosed cancer and the third leading cause of cancer death worldwide in 2020, with approximately 906,000 new cases and 830,000 deaths, and hepatocellular carcinoma (HCC) accounts for about 75–85% of all liver cancers^1^. The vast majority of cases develop in patients with chronic liver diseases, the main risk factors being Hepatitis B Virus (HBV) and Hepatitis C Virus (HCV) infection, alcohol intake and metabolic syndrome^2^. The potential radical treatment for HCC mainly relies on surgical resection, however, the recurrence rate of HCC after hepatectomy within 5 years is as high as 50–70%, which is the main reason for the poor prognosis of HCC^3^. The mechanism of the recurrence of HCC after liver resection is not clear yet, it be related to tumor size, tumor number, AFP levels, tumor differentiation, microvascular invasion(MVI), cirrhosis, positive surgical margin and the metabolic syndrome and so on^4,5^, however, those indicators in daily clinical work is not convenient, and the predictive value of single index has certain limitation, such as AFP levels, poor sensitivity and the prediction is delayed^6^. Therefore, it is necessary to find new combined prognostic indicators to predict the recurrence of HCC after hepatectomy. However, inflammatory and tumor burden have become the focus of the research on the prognosis of HCC.

The mechanism between inflammation and tumor is unclear, there is a lot of basic research found that neutrophils and platelets, monocytes cells and other inflammation cells plays an important role in tumor progression including initiation, malignant conversion, promotion, invasion, and metastasis^7–9^, for example, hepatitis B causes liver cancer, Helicobacter pylori causes stomach cancer, therefore, several prognostic scores such as monocyte lymphocyte count ratio(MLR), platelet-lymphocyte ratio (PLR), and neutrophil–lymphocyte ratio(NLR), systemic immune-inflammation index(SII) was used to predict the prognosis of HCC after liver resection^10–13^, which is being associated with poor prognosis and high recurrence in HCC after liver resection.

Tumor burden score (TBS) was developed by SASAKI as a survival prediction tool for patients undergoing hepatic resection of colorectal liver metastasis (CRLM) based on tumor maximum diameter and number of tumors, and TBS was found to be a good predictor of prognosis in CRML. With the increase of TBS, the survival rate of liver metastasis of colon cancer decreased, and TBS had better predictive ability than traditional tumor size and number^14^. The diameter and size of tumors can be obtained by pathology and imaging, and TBS derived from the two may differ. Subsequently, SASAKI found in another study that TBS obtained based on imaging and pathology had no difference in the predictive value of CRML, and was also superior to tumor diameter and number^15^. Both tumor diameter and number are reliable prognostic indicators for recurrence of HCC after liver resection, which provides evidence that TBS may be a potential prognostic indicator for recurrence of HCC after resection. In order to verify the prognostic value of TBS in HCC patients, scholars from the United States and Italy carried out a prospective study of 24 consecutive HCC patients who participated in ITA, LI and CA (n = 4759). The study found that TBS model could well predict the recurrence and survival of HCC patients. And has the best discrimination and adaptability compared to other continuous or binary variables^16^. Surgical resection of poly-nodular HCC beyond the Milan criteria is controversial, related research found that the prognosis of patients with multiple nodular liver cancer depends on the total tumor burden, to a great extent, for beyond Milan criteria of low-level TBS nodular liver cancer patients, liver resection should be considered^17^. More and more studies use TBS to stratify prognosis of HCC after liver transplantation, liver resection and TACE^18–21^. Obviously, the prognostic value of TBS model in HCC has been fully verified. Although there are few relevant studies, more and more attention has been paid to it.

To our knowledge, there is no report on the prediction of recurrence of HCC after hepatectomy in combination with inflammatory index and TBS. This study for the first time included tumor burden index (TBS) and inflammation index to develop a new predict model for HCC after liver resection. The purpose of this study was to investigate whether the model combination with immune inflammation index and TBS has a better prediction value for recurrence of HCC after liver resect.

## Methods and materials

### Study population and design

**Figure 1:**
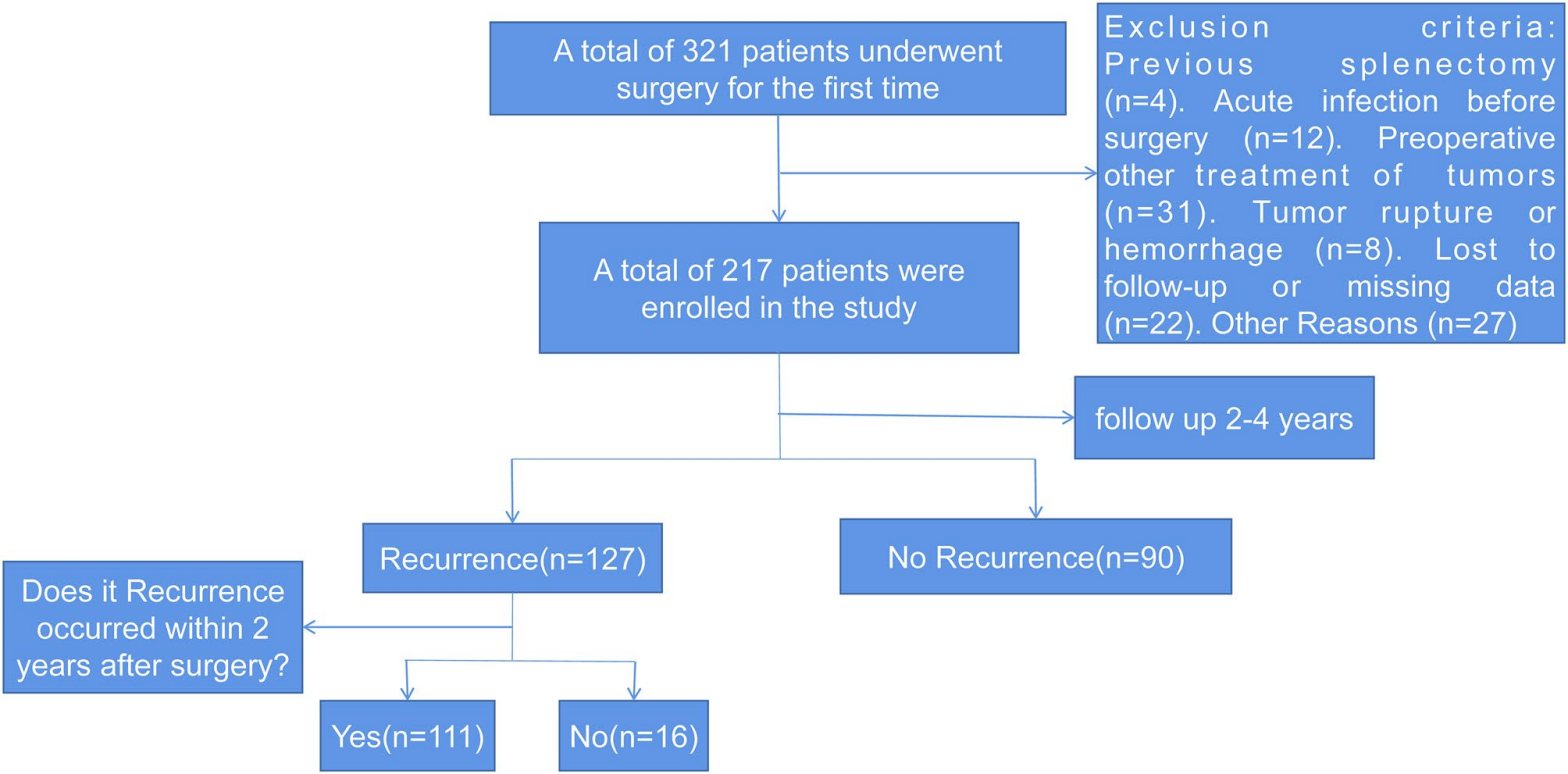
Flow chart for screening patients.

A total of 217 patients who received liver resection for HCC at Xiangya Hospital Central South University met the following criteria and were included in this study (Fig. 1).

*Inclusion criteria* (1) HCC diagnosed by postoperative pathology; (2) Liver function was child–pugh grade A–B; (3) The ECOG PS at 0–2 levels; (4) Perform radical hepatocellular carcinoma resection for the first time to ensure thoroughness of tumor resection (R0 resection) and safety (sufficient volume of functional liver tissue); (5) No distant liver metastasis was found before or during surgery.

*Exclusion criteria* (1) Previous splenectomy; (2) Complicated with acute infectious diseases, such as lung infection and urinary tract infection; (3) Received other preoperative treatments, such as ablation and intervention and so on; (4) Rupture or hemorrhage of the patient's liver cancer before surgery; (5) Patients' clinical data were incomplete or lost to follow-up during follow-up. (6) Complicated with other types of malignant tumors; (7) Due to tumor rupture caused by intraoperative specimen collection, the size and number of tumor could not be obtained by postoperative pathology; (8) Complicated with autoimmune diseases and blood system diseases; (9) Patients died or underwent liver transplantation before the occurrence of tumor recurrence.

### Follow-up and definitions

Blood routine tests and liver function tests of all patients with HCC were obtained by routine blood sampling within 1–2 weeks before liver resection. The maximum diameter and number of tumors were determined according to postoperative pathological examination of patients. MLR = Monocyte/lymphocyte^11^, PLR = platelet/lymphocyte^13^, NLR = Neutrophil /lymphocyte^12^, SII = platelet count × neutrophil count /lymphocyte count^10^, TBS = $\sqrt {a^{2} + b^{2} }$ (a = maximum tumor diameter (cm), b = tumor number)^14^,TTV = 3.14 × 4/3 × (maximum tumor radius in cm)^3^^22,23^. Liver function was assessed with the ALBI score and ALBI grade, which were defined by the ALBI linear predictor. ALBI linear predictor^24^ = (log10bilirubin × 0.66) + (albumin × − 0.085), where bilirubin is in umol/L and albumin in g/L, for ALBI score calculation. Patients were categorised into the ALBI grades by applying cut-off to the linear predictor: grade 1: ≤ − 2.60; grade 2: >  − 2.60 and ≤ − 1.39; and grade 3: > − 1.39. According to the recurrence of patients, the optimal cut-off value of inflammatory indicators and TBS was calculated by ROC curve. The clinical indicators of patients included gender, age, HBV/HCV infection, tumor size, number of tumors, BCLC stage and so on. AFP, PT, APTT, WBC, PLT, ALT, AST, HBV-DNA quantitative were recorded. All of patients underwent preoperative chest radiography, transabdominal color Doppler scanning, abdominal enhanced CT or MRI and stage of HCC by BCLC stage method.

The evaluation of tumors was conducted by conventional US and dynamic contrast enhanced US/computed tomography (CT)/magnetic resonance imaging (MRI) at 1, 3, and 6 months after initial treatment and then at every 6 months. If new lesions were detected in surveillance US or an elevated serum level of AFP > 20 ng/mL, tumor recurrence was suspected and further be confirmed by CEUS/CT/MRI. If tumor recurrence was confirmed, according to economic conditions and will of parents, after evaluation by professional medical team, corresponding treatment will be carried out.

### Statistical analysis

Statistical analysis was performed using IBM SPSS software (version 26.0; SPSS Inc., Chicago, IL, USA), and Graph Pad Prism (version 5.0; USA). Student’s tests were used to analyze the quantitative data. Chi-square tests or Fisher’s exact tests were used to analyze the categorical Variables, Cox proportional hazards regression was used to determine the prognostic factors associated with RFS with univariate and multivariate analyses. The hazard ratio (HR) and 95% confidence interval (CI) were used to describe the relative risk factors. The RFS rates were calculated using the Kaplan–Meier method, and the log‑rank test was used to compare differences among different groups. *P* < 0.05 was considered statistically significant.

### Ethics approval and consent to participate

All methods were carried out in accordance with relevant guidelines and regulations. All experimental protocols were approved by Ethics Committee of Xiangya Hospital of Central South University. Informed consent was obtained from all subjects and/or their legal guardian.

### Consent for publication

Written informed consent for publication was obtained from all participants.

## Result

### Baseline data of the HCC patients

A total of 217 patients were enrolled in this study, including 191 (88.02%) males and 26 (11.98%) females, with an average age of 50.12 ± 12.07 years. Among them, 194 patients (94.47%) were complicated with HBV infection, and no hepatitis C patients were found. 47 (21.66%) patients received regular antiviral therapy (> 6 months) and 119 patients (45.16%) were preoperatively active virus replication (HBV-DNA > 1000 IU/mL). 33 (15.2%) patients had multiple tumors, the mean maximum tumor diameter was 5.93 ± 3.17 cm, 67 (37.89%) patients with AFP > 400 ng/ml, 168 patients with cirrhosis, 172 (79.26%) patients with BCLC grade 0/A and 45 (20.74%) patients with BCLC grade B-C. By the end of follow-up, the median follow-up time is 21.32 ± 14.40 months.127 patients (58.53%) relapsed, among which 111 patients (51.15%) relapsed within 2 years after surgery, most patients have intrahepatic recurrence (Table 1).

**Table 1:** The clinicopathological variables in patients with HCC (n = 217).

Variables	*N* = *217*
Age (years), mean (SD)	50.12 (12.7)
Albumin (g/L), mean (SD)	41.36 (4.36)
WBC (109/L), mean (SD)	5.33 (1.78)
Neutrophil (109/L), mean (SD)	3.26 (1.23)
Lymphocyte (109/L), mean (SD)	1.44 (0.50)
Monocyte (109/L), mean (SD)	0.45 (0.18)
PLT (109/L), mean (SD)	152.37 (66.82)
Total bilirubin (μmol/L), mean (SD)	13.05 (5.78)
Direct bilirubin (μmol/L), mean (SD)	6.10 (2.79)
ALBI (μmol/g), mean (SD)	− 2.81 (0.37)
ALT (U/L), mean (SD)	43.08 (46.09)
AST (U/L), mean (SD)	45.94 (32.94)
PT (s), mean (SD)	13.92 (1.34)
ATPP (s), mean (SD)	35.13 (4.13)
Tumor diameter (cm), mean (SD)	5.93 (3.47)
TBS, mean (SD)	6.10 (3.40)
TTV, mean (SD)	242.73 (423.90)
SII, mean (SD)	389.09 (299.60)
PLR, mean (SD)	114.05 (54.72)
MLR, mean (SD)	0.33 (0.16)
NLR, mean (SD)	2.50 (1.57)
**Gender**	
male	191 (88.02%)
female	26 (11.98%)
**HBV**	
Presence	194 (89.40%)
Absence	23 (10.60%)
**Antiviral therapy**	
Yes	47 (21.66%)
No	170 (78.34%)
**CSPH**	
Yes	41 (18.89%)
No	176 (81.11%)
**ALBI grade**	
I	56 (25.82%)
II	161 (74.18%)
**HBV-DNA**	
≥ 1000 c	119 (54.84%)
< 1000 c	98 (45.16%)
**AFP**	
≥ 400 ng/mL	67 (30.88%)
< 400	150 (69.12%)
**Tumor number**	
Single	184 (84.79%)
Multiple	33 (15.21%)
**Degree of differentiation**	
High and middle	178 (82.03%)
Low	39 (17.97%)
**Satellite nodules**	
Yes	162 (74.65%)
No	55 (25.35%)
**MVI**	
Yes	108 (49.77%)
No	109 (50.23%)
**Cirrhosis**	
Yes	168 (77.42%)
No	49 (22.58%)
**BCLC stage**	
0-A	172 (79.26%)
B-C	45 (20.74%)
Median follow-up period (SD, month)	21.32 (14.40)
Relapsed	127
Within 2 years	111
Beyond 2 years	16
Intrahepatic metastasis	117
Extrahepatic metastasis	10
No relapsed	90

*WBC* white blood cell, *PLT* platelet, *ALT* alanine transaminase, *AST* aspartate trasaminase, *PT* prothrombin time, *APTT* activated partial thromboplastin time, *AFP* alpha-fetoprotein, *MVI* microvascular invasion, *BCLC* Barcelona Clinic LIVER Cancer, *AIBL* albumin-bilirubin grade, *CSPH* clinically significant portal hypertension.

### The cut-off value and area under the curve of the inflammation index and TBS

**Figure 2:**
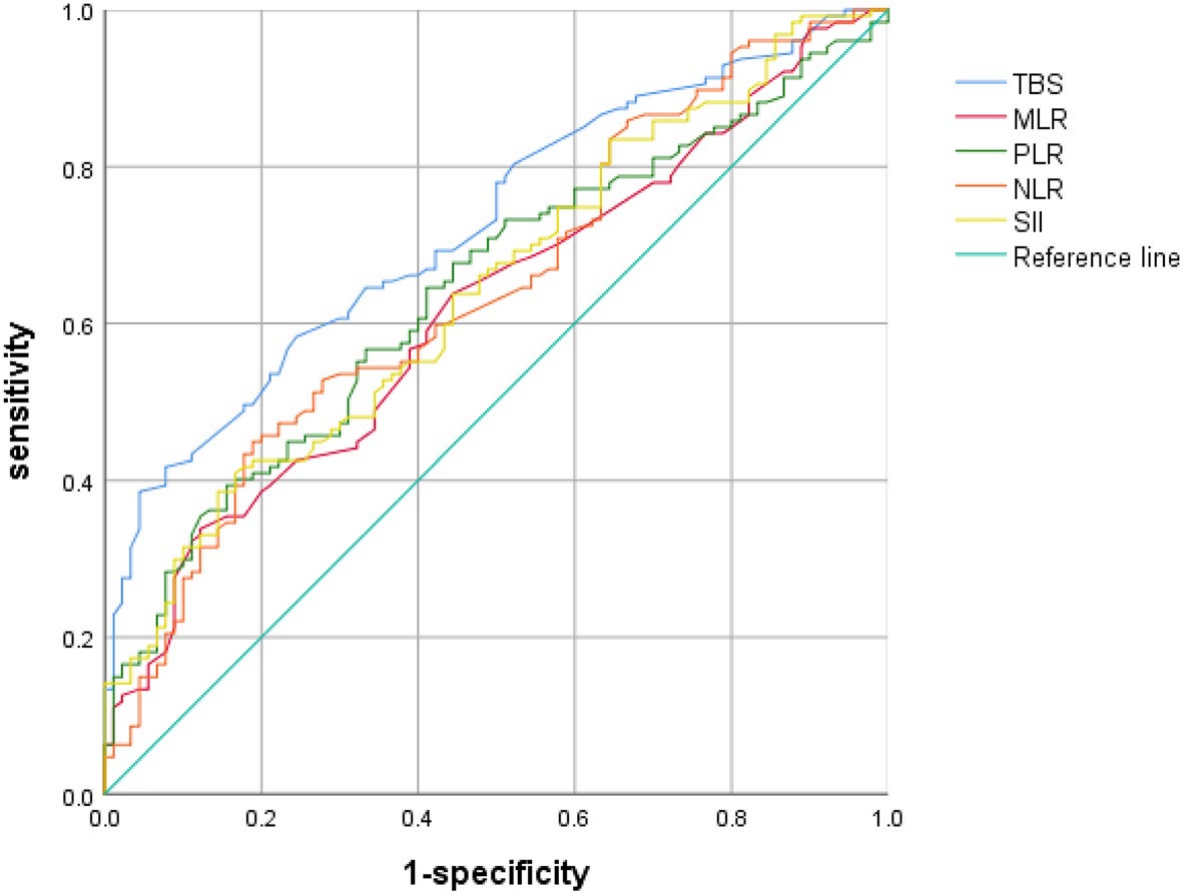
Comparison of the infammatory index and TBS in predicting recurrence.

The ROC curve of patients was drawn according to the preoperative MLR, NLR, PLR, SII and TBS values. The results of ROC curves showed that MLR = 0.39, NLR = 2.63, PLR = 134, SII = 428 and TBS = 8.06 were the optimal cut-off values (Table 2). By based on the optimal cut-off value, patients with less than or equal to the cut-off value were assigned to the low-level group, and patients with greater than the cut-off value were assigned to the high-level group. The AUC of SII, PLR, NLR, MLR and TBS were 0.643, 0.642, 0.642, 0.618 and 0.724, respectively. The sensitivity of SII, MLR, PLR, NLR and TBS were 38.6%, 32.3%, 39.4.9%, 44.9% and 38.6%, respectively, and the specificity was 85.6%, 88.9%, 84.4%, 81.1% and 95.6%, respectively. By comparing AUC (Fig. 2), we found that TBS was superior to other inflammatory indicators in predicting recurrence of HCC patients after operation. Moreover, among the four inflammatory indicators, SII, PLR and NLR had similar prognostic value for postoperative recurrence of HCC, but they were all superior to MLR.

**Table 2:** The cut-of value and area under the curve of the inflammation index and TBS.

Factor	Cut-off value	AUC	Sensitivity (%)	Specifcity (%)	Youden index	95%CI of AUC
SII	428	0.643	38.6	85.6	0.242	0.570–0.716
MLR	0.39	0.618	32.3	88.9	0.212	0.544–0.692
PLR	134	0.642	39.4	84.4	0.238	0.569–0.715
NLR	2.63	0.642	44.9	81.1	0.260	0.568–0.716
TBS	8.06	0.724	38.6	95.6	0.342	0.658–0.790

*AUC* area under curve, *CI* confidence interval, *MLR* monocyte to lymphocyte ratio, *PLR* platelet-to-lymphocyte ratio, *SII* systemic immune-inflammation index, *NLR* neutrophil to lymphocyte ratio, *TBS* tumor burden score.

### Comparison of TBS value with maximum tumor diameter, number of tumors and TTV values in prognostic accuracy

**Figure 3:**
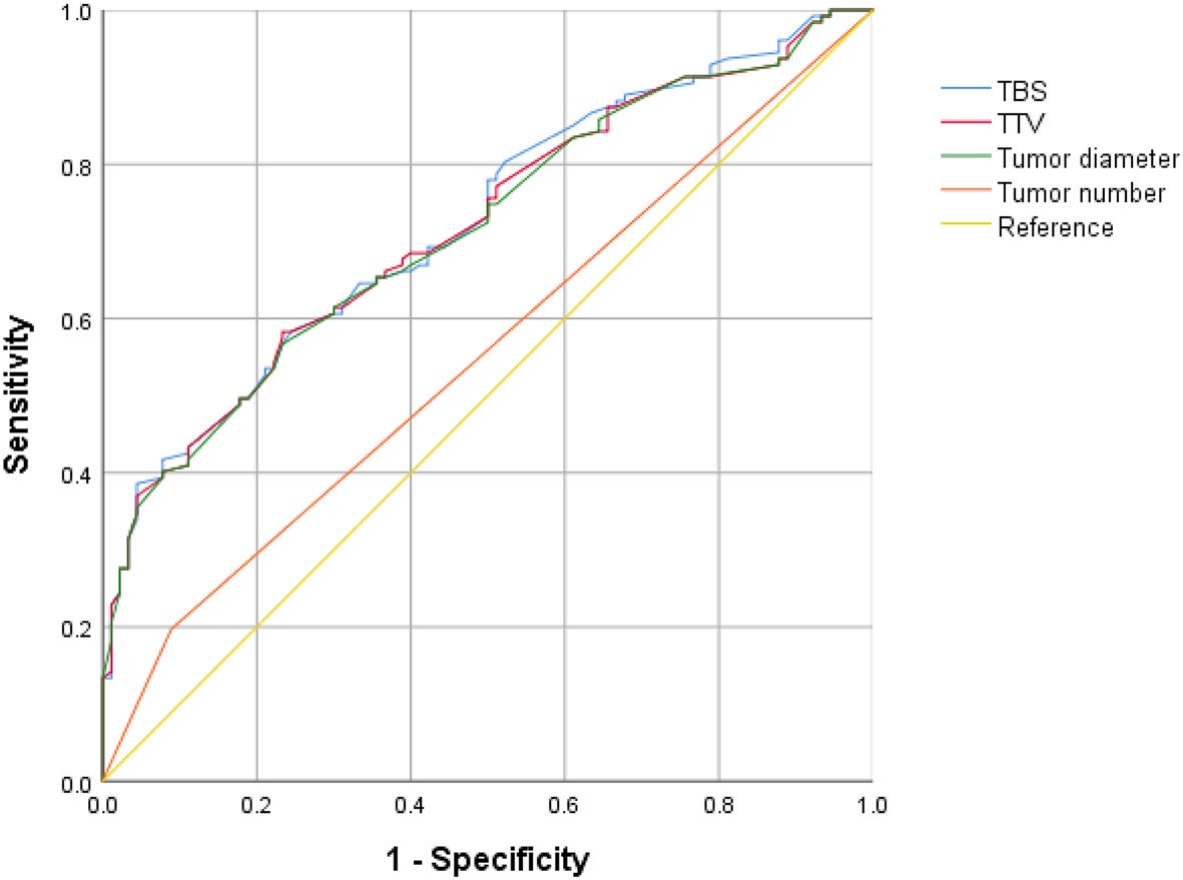
Comparison of TBS, TTV, tumor diameter and tumor number in predicting recurrence.

Then we drew ROC curves for TBS values, maximum tumor diameter and tumor number and TTV values of all HCC patients. According to their ROC curves, AUC of TBS, tumor diameter, tumor number and TTV values were 0.724, 0.715, 0.554, 0.719 respectively (Fig. 3). By comparing the AUC of those indicators, the predictive value of TBS for postoperative recurrence of HCC was better than that of tumor diameter, tumor number and TTV values. This advantage may be more pronounced in a large sample of patients with multiple tumors.

### Univariate and multivariate analysis of recurrence‑free survival

Univariate Cox analysis was performed for gender, age, albumin, total bilirubin, ALBI, TBS values ,TTV values, ALT, AST, PT, APTT, tumor diameter, number of tumors, AFP, degree of differentiation, HBV infection, HBV-DNA quantification, cirrhosis, MVI, satellite nodules, BCLC stage, SII, NLR, MLR, PLR and TBS group, We found TBS values (*P* < 0.001), TTV values (*P* < 0.001), AFP > 400 ng/mL (*P* < 0.010), tumor diameter (*P* < 0.001), tumor number (*P* < 0.001), poor/moderate differentiation (*P* < 0.001), MVI (*P* < 0.001), satellite nodule (*P* < 0.001), BCLC B-C stage (*P* < 0.001), SII > 428 (*P* < 0.001), NLR > 2.63 (*P* < 0.001), MLR > 0.39 (*P* < 0.001), PLR > 134 (*P* < 0.001) and TBS > 8.06 (*P* < 0.001) are the possible factors for recurrence of HCC after liver resection (Table 3).

**Table 3:** Univariate analysis of RFS.

Factors	HR	95% CI	*P* value
Sex (male/female)	0.941	(0.972–1.001)	0.821
Age (years)	0.987	(0.972–1.001)	0.078
Antiviral therapy (yes/no)	1.337	(0.857–2.086)	0.200
CSPH (yes/no)	1.531	(0.940–2.495)	0.087
ALBI (μmol/g)	1.471	(0.912–2.371)	0.116
ALBI grade (I/II)	0.884	(0.597–1.310)	0.539
Albumin (g/L)	0.967	(0.928–1.008)	0.160
Total bilirubin (umol/L)	1.008	(0.978–1.039)	0.607
ALT (U/L)	1.000	(0.996–1.003)	0.857
AST (U/L)	1.005	(1.000–1.009)	0.065
APTT (s)	1.156	(0.772–1.731)	0.480
PT (s)	1.032	(0.722–1.475)	0.863
HBV-DNA (≥ 1000/ <1000 c)	1.076	(0.759–1.525)	0.680
Tumour size	1.154	(1.105–1.204)	< 0.001
Tumour number (solitary/multiple)	1.868	(1.204–2.898)	0.005
TTV values	1.001	(1.000–1.001)	< 0.001
TBS values	1.159	(1.110–1.120)	< 0.001
AFP (≥ 400/ < 400 ng/mL)	1.681	(1.122–2.334)	0.010
Differentiation (poor/moderate and well)	3.113	(2.008–4.642)	< 0.001
HBV infection (yes/no)	1.166	(0.643–2.114)	0.606
Liver cirrhosis (yes/no)	0.820	(0.544–1.237)	0.345
MVI (yes/no)	3.053	(2.114–4.410)	< 0.001
satellite nodule (yes/no)	2.424	(1.677–3.504)	< 0.001
BCLC stage (B-C/0-A)	3.327	(2.263–4.893)	< 0.001
SII (high/low)	2.371	(1.654–3.397)	< 0.001
PLR (high/low)	2.162	(1.510–3.098)	< 0.001
NLR (high/low)	2.273	(1.599–3.231)	< 0.001
MLR (high/low)	2.164	(1.489–3.146)	< 0.001
TBS (high/low)	3.714	(2.560–5.389)	< 0.001

*ALT* alanine transaminase, *AST* aspartate trasaminase, *PT* prothrombin time, *APTT* activated partial thromboplastin time, *AFP* alpha-fetoprotein, *MVI* microvascular invasion, *BCLC* Barcelona Clinic LIVER Cancer, *MLR* monocyte to lymphocyte ratio, *PLR* platelet-to-lymphocyte ratio, *SII* systemic immune-inflammation index, *NLR* neutrophil to lymphocyte ratio, *TBS* tumor burden score, *HR* hazard ratio, *AIBL* albumin-bilirubin grade, *CSPH* clinically significant portal hypertension.

Considering TBS calculated by tumor diameter and tumor number, Multivariate COX risk regression analysis was performed after tumor size and diameter were removed. Only MVI (HR = *P* = 0.005), satellite nodule (*P* = 0.017), BCLC B-C stage (*P* = 0.013), NLR > 2.63 (*P* = 0.013), TBS > 8.06 (*P* < 0.017) were independent risk factors for postoperative recurrence of HCC (Table 4).

**Table 4:** Multivariate analyses for RFS.

Factors	HR	95% CI	*P* value
AFP (≥ 400/ < 400 ng/mL)			0.213
Differentiation (poor/moderate and well)			0.091
MVI (yes/no)	1.806	1.190–2.740	0.005
Satellite nodule (yes/no)	1.661	1.094–2.523	0.017
BCLC stage (B-C/0-A)	1.801	1.313–2.868	0.013
SII (high/low)			0.389
PLR (high/low)			0.244
NLR (high/low)	1.928	1.148–3.240	0.013
MLR (high/low)			0.446
TBS (high/low)	1.752	1.106–2.275	0.017

*AFP* alpha-fetoprotein, *MVI* microvascular invasion, *BCLC* Barcelona Clinic Liver Cancer, *MLR* monocyte to lymphocyte ratio, *PLR* platelet-to-lymphocyte ratio, *SII* systemic immune-inflammation index, *NLR* neutrophil to lymphocyte ratio, *TBS* tumor burden score.

### The prognostic value of combined index (NLR–TBS) model for HCC

**Figure 4:**
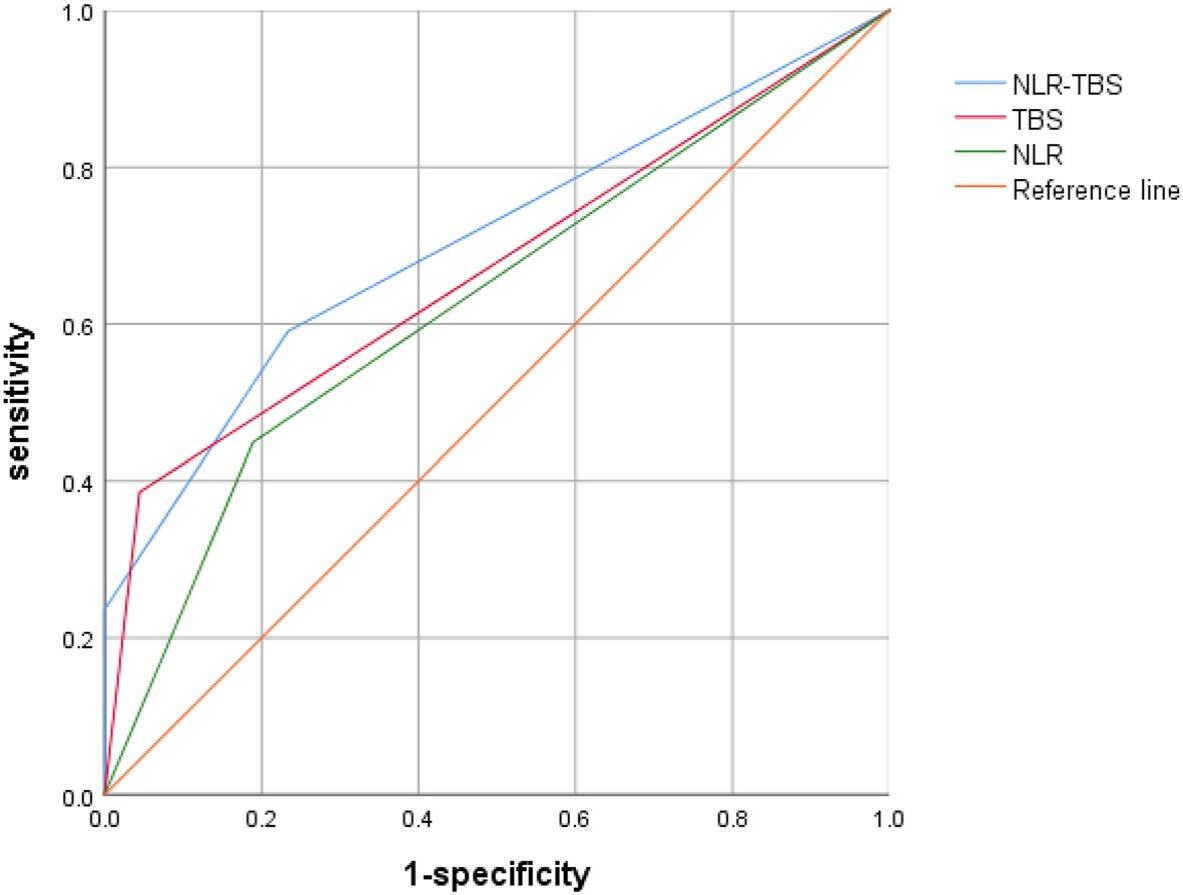
Comparison of NLR–TBS group, TBS group and NLR group in predicting recurrence.

**Figure 5:**
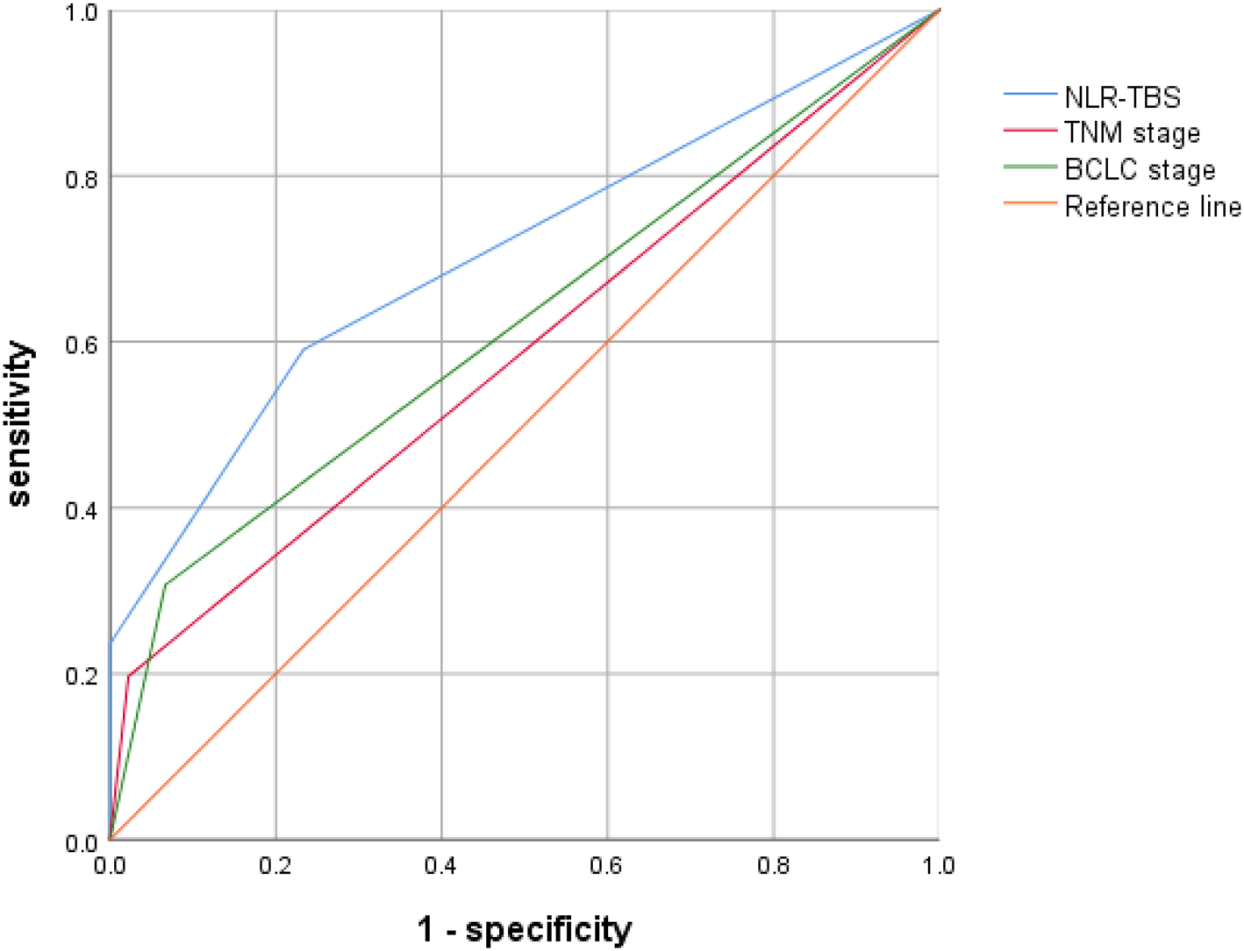
Comparison of NLR–TBS group, TNM stage and BCLC stage in predicting recurrence.

**Figure 6:**
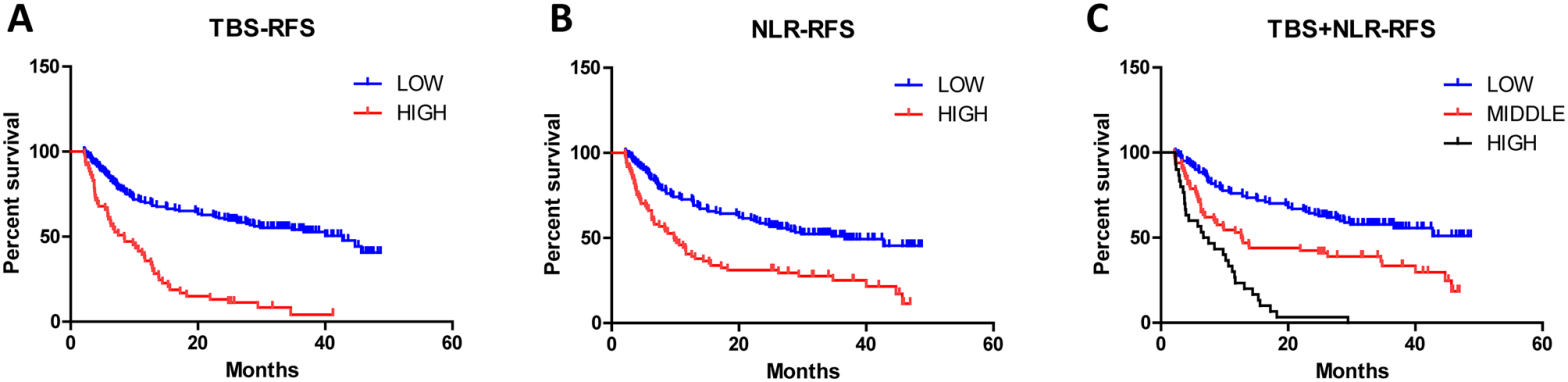
(**A**) Recurrence-free survival curve of patients with high TBS (n = 53) and low TBS (n = 164).The median RFS times in the high TBS group and the low TBS group were 11.61 months and 30.98 months (*P* = 0.001). (**B**) Recurrence-free survival curves of patients with high NLR (n = 74) and low NLR (n = 143).The median RFS times in the high NLR group and the low NLR group were 18.12 months and 30.63 months (*P* = 0.001). (**C**) Recurrence-free survival curves of low-risk (n = 121), middle-risk (n = 66) and high-risk group (n = 30) patients. The median RFS times in the low-risk group, middle-risk group and high-risk group patients were 33.03 months, 22.16 months and 8.07 months (*P* = 0.001).

The optimal inflammatory immune index (NLR) and TBS obtained in this study were combined to stratify the risk of postoperative recurrence of HCC: low risk group (TBS ≤ 8.06 and NLR ≤ 2.63), middle risk group (TBS > 8.06 and NLR ≤ 2.63 or TBS ≤ 8.06 and NLR > 2.63), high risk group (TBS > 8.06 and NLR > 2.63); According to the recurrence of patients, ROC curves were drawn for TBS group, NLR group and NLR–TBS groups (Fig. 4). Areas under ROC curves of TBS, NLR and NLR–TBS groups were as follows: 0.671, 0.630, 0.762, according to the area under the ROC curve, the prognostic value of NLR–TBS for postoperative recurrence of HCC was superior to the single index of NLR and TBS grouping. Finally, the established NLR-TBS prediction model was compared with the traditional HCC stage system (BCLC stage, TNM stage). By drawing ROC curves, the areas under ROC curves of NLR–TBS model, TNM and BCLC stages were 0.762, 0.587 and 0.620 (Fig. 5), respectively. The prognostic value of NLR–TBS model for postoperative recurrence of liver cancer was better than that of TNM stage and BCLC stage. Recurrence risk survival curves of TBS (Fig. 6A), NLR (Fig. 6B) and TBS + NLR (Fig. 6C) were drawn according to the recurrence situation of patients.

## Discussion

Recurrence of HCC after surgical resection is caused by multiple factors, tumor-associated inflammation and TBS reflects tumor morphological characteristics and tumor burden, however, there has been no study on the relationship between combined inflammatory markers and TBS of postoperative recurrence of HCC. Patients in this study were followed up for a short time, but each non-recurrent patient was followed for at least 2 years. Almost all patients survived after the follow-up, and most patients would receive different treatment after relapse, which would affect the overall survival (OS) of patients. Therefore, this study only studied postoperative recurrence of patients. We found that the follow-up peak of HCC recurrence was mainly within 2 years after surgery, and the 2-year cumulative recurrence rate in this study was 51.15%. Among all patients with recurrence (n = 126), 111 patients (61.90%) had recurrence within 2 years after surgery, so it can roughly reflect patients' early postoperative recurrence, which was roughly the same as the results of previous studies^3^.

In this study, we conducted a univariate and multivariate risk analysis on postoperative recurrence of HCC. Only MVI (*P* = 0.004), satellite nodule (*P* = 0.021), BCLC B-C stage (*P* = 0.010), NLR > 2.63 (*P* = 0.013) and TBS > 8.06 (*P* = 0.010) were independent risk factors for postoperative recurrence of HCC, we found that only high-level NLR(NLR > 2.63) was an independent risk factor among various inflammatory indicators, suggesting that the predictive value of NLR in immune inflammation index was better than that of SII, PLR and MLR for HCC after resection, which may be due to the following reasons:1.High level NLR reflects relatively depleted lymphocytes in the blood and tumor, and depleted lymphocytes are associated with poor cancer-specific survival by impairing the host immune response against tumor cells^25^. Lymphocytes have a role in cytotoxic cell death and in producing cytokines to inhibit tumor cells^26^. Elevated NLR is associated with high infiltration of TAMs and high production of inflammatory cytokines, such as interleukin (IL)-6, IL-8, and IL-17, which promote systemic neutrophilia^27,28^. VEGF is considered as a reservoir which promotes angiogenesis and tumor grow^29^. This suggests that the negative outcomes in patients with elevated NLR are mediated by an inflammatory microenvironment, rather than a single pathway. HCC patients often complicated by chronic hepatitis infection, liver cirrhosis and portal hypertension which can cause hyperplenism, hyperplenism can cause a variety of blood cells decreased, the decrease in the number of platelets is most obvious, although this study had splenectomy patients was excluded, but there are still many patients with cirrhosis of the liver and hyperplenism, low platelet count in these patients. As a result, the prognostic effects of SII and PLR containing platelet parameters in liver cancer are not reliable, which explains the controversy over whether high level of SII is a protective factor or a high-risk factor for the prognosis of liver cancer after surgery in various studies^10,30,31^. The contents of lymphocytes and monocytes contained in MLR are low in blood, and there is no significant difference between tumor patients and normal patients, resulting in low sensitivity of MLR in predicting postoperative recurrence of patients.

According to the previous study, TBS is an excellent prognostic indicator, and the calculation of TBS requires the maximum diameter and number of tumors. Since all patients in this study underwent surgery, and most patients had a single large tumor, the calculated TBS value was close to tumor max diameter value. The AUC value of TBS is still higher than the AUC value of tumor diameter and tumor number. In a large sample of patients with multiple tumors, TBS may be more accurate than tumor diameter and tumor number in predicting recurrence of HCC after liver resection, which requires further study to verify. In addition, TBS has great potential in predicting the prognosis of other multiple solid tumors, but there are also some controversies and problems to be solved in TBS: 1.For preoperative unable to obtain pathological tumor size and number of patients with HCC, such as radio-frequency ablation or unresectable liver cancer, the calculation of TBS can only rely on preoperative imaging, and relying on the imaging for tumor size and quantity has some limitations, first of all, the tumor diameter measurement of subjectivity, to a large extent depends on the physician of the selected image measuring plane, Second, the number of tumors are difficult to determine by imaging, especially for some small nodular lesions in patients with cirrhosis, qualitative is relatively difficult, although study have shown based on the imaging and postoperative pathological TBS no statistical difference^15^, but we are saved in question, there's more in the future research is needed to confirm it. This will affect the applicability of TBS model.2. In the past, there is a study put forward the tumor volume (TTV)^32^, as is also the clinical assessment of tumor burden index, have been a number of studies prove its reliability in HCC^33–35^. Preliminarily compared the predictive value of the two variables for postoperative recurrence of liver cancer. Considering that there is no unified standard for the definition of the high and low levels of the two variables, this study took the two variables as continuous variables and compared their predictive performance with the value of AUC. We found that the predictive value of TBS for postoperative recurrence of HCC was better than TTV, but this need more studies to prove it and we're going to use 3D printing to calculate liver volume in the future. 3. There are still few studies on TBS, and more studies with bigger data are needed to prove its reliability.

In this study, NLR combined with TBS was used for the first time to predict postoperative recurrence of HCC. By combining a systemic inflammatory index and a tumor burden index that reflected inflammatory microenvironment and tumor burden, NLR–TBS model was constructed to group the risk of postoperative recurrence of HCC. By comparison of ROC curves, we found that NLR–TBS model had a better predictive function than a single index. All of patients in the high-risk group (n = 30) had postoperative recurrence, 45 patients in the middle-risk group (n = 66) had postoperative recurrence, and 52 patients in the low-risk group (n = 121) had postoperative recurrence. The median RFS in the high-risk group, the middle-risk group and the low-risk group were 8.72 months, 22.16 months and 33.03 months, respectively. NLR and TBS contain a number of prognostic factors, which in this study demonstrated very good predictive ability of postoperative recurrence of liver cancer. NLR and TBS are not only superior to prediction of single index, but also superior to traditional TNM stage and BCLC stage models. They are very convenient, easy and efficient to obtain clinically before surgery, and also applicable to patients with negative AFP. This will greatly increase the clinical use, and can be used as a supplementary tool to help increase the follow-up rate of high-risk patients. For HCC patients with high recurrence risk, earlier and more aggressive treatment can be carried out, including preoperative TACE, postoperative targeted therapy, and other comprehensive treatment, to improve the prognosis of patients and prolong the survival time.

Our study also had some limitations: 1. Our study was a single-center retrospective study with a small sample size and short postoperative follow-up time, which may have various biases. Therefore, multi-center, large-sample and time-span studies are needed to confirm the prognostic value of immune inflammatory indicators, TBS and their combination; 2. Based on the differences in sample inclusion criteria, the cut-off value of inflammation index and TBS varies in different studies. How to obtain a unified level value for clinical use is an urgent problem. 3. The NLR–TBS prediction model established by us lacks external data to verify it, and more internal and external data are needed to verify it in the future.

### Inclusion

We found that NLR and TBS should be good prognostic factor for HCC after liver resection. NLR–TBS model based the best inflammatory index and TBS. The prediction performance of NLR–TBS model not only better than NLR and TBS, but also better than BCLC and TNM stage models.

## Data Availability

All data generated or analysed during this study are included in this published article.
